# Biological Profile of Ferulic Acid-Based Thiazolidin-4-One Derivatives: Toxicity, Inflammation, and Oxidative Stress Biomarkers

**DOI:** 10.3390/molecules31152646

**Published:** 2026-07-29

**Authors:** Maria Dragan, Andreea-Teodora Iacob, Cornelia Mircea, Catalina Daniela Stan, Oana-Maria Dragostin, Alin-Viorel Focșa, Diana Tatarciuc, Ruxandra-Teodora Stan, Mihaela Poroch, Lenuta Profire

**Affiliations:** 1 Department of Pharmaceutical Sciences II, Faculty of Pharmacy, Grigore T. Popa University of Medicine and Pharmacy Iasi, 16 University Street, 700115 Iasi, Romania; maria.wolszleger@umfiasi.ro (M.D.); cornelia.mircea@umfiasi.ro (C.M.); alin-viorel.focsa@umfiasi.ro (A.-V.F.); 2 Department of Pharmaceutical Sciences I, Faculty of Pharmacy, Grigore T. Popa University of Medicine and Pharmacy Iasi, 16 University Street, 700115 Iasi, Romania; lenuta.profire@umfiasi.ro; 3 Research Centre in the Medical-Pharmaceutical Field, Faculty of Medicine and Pharmacy, “Dunărea de Jos” University of Galati, 800201 Galati, Romania; oana.dragostin@ugal.ro; 4 Department of Internal Medicine I, Faculty of Medicine, Grigore T. Popa University of Medicine and Pharmacy Iasi, 16 University Street, 700115 Iasi, Romania; diana.tatarciuc@umfiasi.ro; 5 Department of Implantology, Removable Prosthodontics and Dental Technology, Faculty of Dental Medicine, Grigore T. Popa University of Medicine and Pharmacy Iasi, 16 University Street, 700115 Iasi, Romania; ruxandra-teodora.stan@umfiasi.ro; 6 Department of Preventive Medicine and Interdisciplinarity, Faculty of Medicine, Grigore T. Popa University of Medicine and Pharmacy Iasi, 16 University Street, 700115 Iasi, Romania; boanca.mihaela@umfiasi.ro

**Keywords:** thiazolidin-4-one, toxicity determination, anti-inflammatory action, ferulic acid derivatives

## Abstract

Since inflammation is a fundamental process involved in tissue injury and repair, the evaluation of the anti-inflammatory potential of novel compounds is of considerable importance. Acute toxicity evaluation in Swiss albino mice revealed that the synthesized thiazolidin-4-one derivatives of ferulic acid possess moderate toxicity. The in vivo biological activity of synthesized thiazolidin-4-one derivatives (**1a**–**j**) was assessed through a model of acute inflammation induced by carrageenan in rats and in a chronic inflammation model induced in rats using the granuloma test. The compounds exhibited significant anti-inflammatory effects, with maximum activity observed 24 h after administration, suggesting a prolonged duration of action. In the acute inflammation model, compound **1g** exhibited the highest anti-inflammatory activity, achieving 97.43% inhibition of inflammatory edema after 24 h. In the chronic inflammation model, all derivatives reduced granulation tissue formation, indicating inhibition of the proliferative component of inflammation, with compound **1e** showing the highest inhibition (79.85%). Safety evaluation was complemented by biochemical and hematological investigations performed on blood samples collected from the experimental animals. Hepatic function was assessed by measuring serum aspartate aminotransferase (AST) and alanine aminotransferase (ALT), together with renal and hematological parameters, revealing no significant toxicity. In addition, biochemical analysis of liver homogenates demonstrated modulation of oxidative stress markers, including malondialdehyde (MDA), superoxide dismutase (SOD), and catalase (CAT) activities, indicating that the investigated thiazolidin-4-one derivatives do not induce marked oxidative imbalance under the experimental conditions. Overall, compound **1g** emerged as the most promising derivative based on its balanced anti-inflammatory efficacy and favorable oxidative stress profile.

## 1. Introduction

Inflammation is a protective biological response that contributes to tissue repair and host defense [1]. However, persistent or dysregulated inflammation is implicated in the pathogenesis of numerous chronic disorders, including cardiovascular diseases, diabetes mellitus, cancer, rheumatoid arthritis, and neurodegenerative diseases [2,3,4,5,6]. The inflammatory response is closely associated with oxidative stress and excessive production of reactive oxygen species (ROS), which further amplify tissue injury and disease progression [7,8]. Therefore, agents that combine a favorable redox safety profile and anti-inflammatory properties are of great interest in the discovery of novel therapeutic strategies.

Ferulic acid, a widespread phenolic compound present in plant cell walls, is known for its strong antioxidant capacity, radical scavenging properties, and multiple pharmacological effects [9,10,11]. Both ferulic acid and its derivatives have been reported to protect against oxidative-stress-induced tissue injury [12], to modulate inflammatory pathways, and to exert beneficial effects in conditions such as diabetes [13], cancer [14,15] and neurodegenerative diseases [16,17]. These findings have encouraged the development of numerous ferulic acid derivatives designed to improve pharmacological efficacy, bioavailability, and biological activity while preserving the favorable properties of the parent molecule [18].

On the other hand, thiazolidin-4-ones represent a privileged class of heterocyclic compounds extensively studied in medicinal chemistry. Their structural scaffold has been associated with a wide spectrum of biological activities, including antimicrobial [19,20], anticancer [21,22], antitubercular [23], antidiabetic [24], anti-Alzheimer [25,26], anticonvulsant [27] and especially anti-inflammatory effects [28,29,30]. Structural versatility and ease of functionalization make thiazolidin-4-one derivatives attractive candidates for drug design. Several reports suggest that substitution patterns on the thiazolidinone ring can significantly modulate pharmacological activity, particularly in the context of anti-inflammatory potential [30,31].

Ferulic acid has been extensively reported to exert anti-inflammatory activity by modulating oxidative stress-sensitive signaling pathways, particularly through activation of the nuclear factor erythroid 2-related factor 2/heme oxygenase-1 (Nrf2/HO-1) axis and suppression of nuclear factor kappa B (NF-κB)-mediated inflammatory responses, thereby reducing the production of pro-inflammatory cytokines and inflammatory mediators [9,32]. In contrast, thiazolidin-4-one derivatives have been associated with the modulation of multiple inflammatory targets, including cyclooxygenase-2 (COX-2), 5-lipoxygenase (5-LOX), inducible nitric oxide synthase (iNOS), and NF-κB signaling pathways [33,34]. Based on these complementary mechanisms, the rational combination of the ferulic acid scaffold with the pharmacologically versatile thiazolidin-4-one nucleus was considered a promising strategy for the development of hybrid molecules that combine anti-inflammatory activity with a favorable redox safety profile. The selected aromatic substituents were chosen to provide derivatives with diverse electronic and steric characteristics, allowing an initial exploration of the influence of aromatic substitution on biological activity and the establishment of initial structure–activity relationship (SAR) trends. In the present study, we investigated the biological profile of a series of ferulic acid-derived thiazolidin-4-one compounds previously synthesized and structurally characterized by our group [35]. Our previous studies demonstrated their preliminary antioxidant activity using in vitro DPPH and ABTS radical-scavenging assays [35] and their anti-inflammatory potential using in vitro protein denaturation and membrane stabilization assays [36]. The present work provides the first comprehensive in vivo evaluation of this series. The study comprised acute toxicity assessment, in vivo anti-inflammatory evaluation in acute and chronic inflammation models, together with biochemical, hematological, and oxidative stress assessments, providing a comprehensive toxicological and pharmacological profile.

## 2. Results

**Figure 1:**
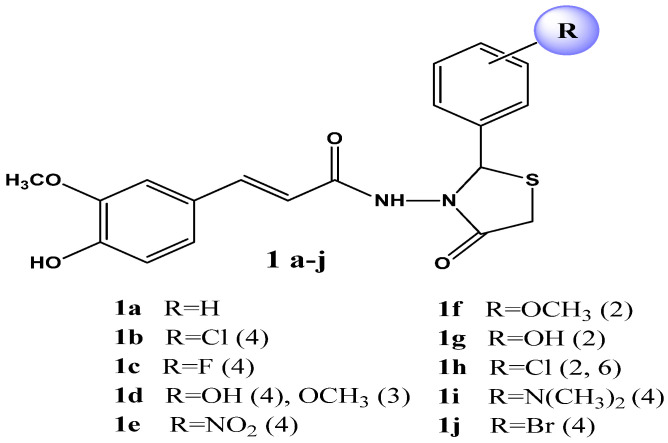
Chemical structures of the ferulic acid-based thiazolidin-4-one derivatives (**1a**–**j**).

The previously synthesized ferulic acid-derived thiazolidin-4-one derivatives (**1a**–**j**) [35] were subjected to a comprehensive in vivo pharmacological evaluation. For clarity, the general chemical structure of the investigated compounds is presented in Figure 1.

### 2.1. Determination of Acute Toxicity

The acute toxicity of the **1a**–**j** derivatives was evaluated by determining the LD_50_ values (Table 1). Depending on the value obtained for LD_50_, pharmaceutical substances can be classified into six categories: supertoxic, extremely toxic, very toxic, moderately toxic, slightly toxic or practically non-toxic.

Considerable differences in toxicity were observed among the investigated compounds. Derivatives **1e** (4-NO_2_; LD_50_ = 5000 mg/kg) and **1c** (4-F; LD_50_ = 4812.5 mg/kg) exhibited the lowest acute toxicity, indicating the most favorable safety profile within the investigated series. In contrast, compounds **1d** (4-OH, 3-OCH_3_; LD_50_ = 925 mg/kg) and **1a** (H; LD_50_ = 987.5 mg/kg) showed the highest toxicity, whereas the remaining derivatives displayed intermediate LD_50_ values ranging from 1250 to 2875 mg/kg. Overall, all investigated derivatives were classified as moderately toxic according to the obtained LD_50_ values. The observed variability in LD_50_ values suggests that both the electronic nature and the position of the aromatic substituents influence the acute toxicity of this series, an aspect further discussed in relation to the structure–activity relationship.

### 2.2. Determination of In Vivo Anti-Inflammatory Potential

#### 2.2.1. Acute Inflammation Model Induced with Carrageenan in Rats

**Figure 2:**
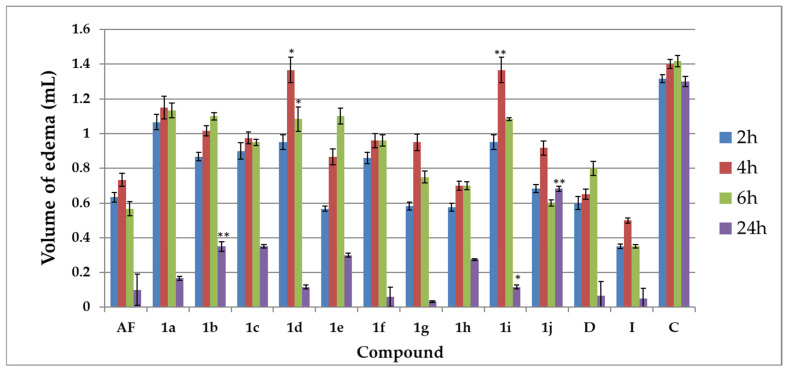
Time-course of carrageenan-induced paw edema in rats treated with compounds **1a**–**j** (administered at 1/10 of their respective LD_50_ values), ferulic acid (AF), diclofenac sodium (D), indomethacin (I), and the vehicle (Tween 80) control group (C). Data are presented as mean ± SD (*n* = 6). * *p* < 0.05 and ** *p* < 0.01 compared with the vehicle (Tween 80) control group.

The anti-inflammatory activity of the synthesized thiazolidin-4-one derivatives (**1a**–**j**) was evaluated in the carrageenan-induced paw edema model in rats and compared with the reference anti-inflammatory drugs diclofenac sodium (D) and indomethacin (I). A time-dependent anti-inflammatory effect was observed for all investigated derivatives (Figure 2, Table 2). A reduction in paw edema volume indicates a greater anti-inflammatory effect of the tested compounds. The strongest anti-inflammatory activity after 24 h was observed for compounds **1g** (97.43%, R = 2-OH) and **1f** (95.38%, R = 2-OCH_3_). Their inhibitory values slightly exceeded those of diclofenac (94.87%) and indomethacin (96.15%), suggesting a prolonged anti-inflammatory effect. Compounds **1i** (94.87%, R = 4-N(CH_3_)_2_) and **1d** (91.02%, R = 4-OH, 3-OCH_3_) also exhibited inhibition values numerically comparable to those of the reference drugs. In contrast, compound **1h** (R = 2,6-di Cl) showed a more consistent inhibitory profile during the early stages of inflammation, maintaining inhibition values close to 50%.

#### 2.2.2. Anti-Inflammatory Effect on Chronic Inflammation Model

The anti-inflammatory activity of the synthesized ferulic acid-derived thiazolidin-4-one derivatives (**1a**–**j**) was evaluated using the cotton pellet-induced granuloma model, which allows the assessment of both the transudative and proliferative phases of chronic inflammation.

*Transudative phase*—During the transudative phase (Table 3), all investigated derivatives reduced exudate formation compared with the control group. The highest inhibitory activity was observed for compounds **1a** (42.47%, R=H) and **1c** (41.35%, R=4-F), whose inhibition values were numerically comparable to that of diclofenac sodium (42.69%) and higher than that of indomethacin (34.48%). Compounds **1g** (39.86%, R=2-OH) and **1e** (37.77%, R=4-NO_2_) also demonstrated pronounced inhibitory activity, whereas derivatives **1b** (R=4-Cl), **1f** (R=2-OCH_3_), and **1j** (R=4-Br) exhibited comparatively lower inhibition values (13.81%, 17.62% and 18.59%).

*Proliferative phase*—During the proliferative phase (Table 4), all synthesized derivatives inhibited granuloma formation to different extents. The strongest activity was observed for compound **1e** (79.85%, R = 4-NO_2_), followed by compounds **1j** (74.49%, R = 4-Br), **1d** (73.35%, R = 4-OH, 3-OCH_3_), and **1c** (71.05%, R = 4-F). Compound **1e** exhibited an inhibition value numerically close to that of indomethacin (81.25%), whereas all investigated derivatives showed lower inhibition than diclofenac (89.49%). In contrast, compound **1b** (2.05%, R=4-Cl) displayed the lowest inhibitory activity, indicating a markedly weaker effect on the proliferative component of chronic inflammation.

### 2.3. Evaluation of Some Biochemical and Hematological Parameters in a Chronic Inflammation Model

#### 2.3.1. Biochemical Parameters for Assessing Liver Function

The liver function parameters, including aspartate aminotransferase (AST), alanine aminotransferase (ALT), lactate dehydrogenase (LDH), total bilirubin, and direct bilirubin, determined after treatment with the synthesized derivatives are presented in Table 5. Among the investigated compounds, derivative **1b** (R = 4-Cl) exhibited the most favorable hepatic profile, showing the lowest AST (124.5 IU/L) and LDH (617 IU/L) activities together with low ALT values (50.5 IU/L). In contrast, compound **1e** (R = 4-NO_2_) produced the highest AST and ALT activities (545 IU/L and 130.5 IU/L), whereas the highest LDH activity was observed for compound **1c** (3778 IU/L, R = 4-F). Total and direct bilirubin concentrations remained within a relatively narrow range across all experimental groups, indicating preserved hepatic metabolic and excretory function.

#### 2.3.2. Biochemical Parameters of Lipid Profile

The lipid profile parameters determined after treatment with the synthesized derivatives are presented in Table 6. Differences among the investigated compounds were observed for all lipid parameters. Most derivatives exhibited LDL cholesterol values numerically comparable to those of the control groups. In contrast, total cholesterol and triglyceride concentrations showed greater variability. Compound **1e** (R = 4-NO_2_) displayed the highest triglyceride concentration (142.5 mg/dL), whereas compounds **1d** (R = 4-OH, 3-OCH_3_) and **1g** (R = 2-OH) exhibited the lowest total cholesterol values (50 and 51.5 mg/dL, respectively). The highest HDL cholesterol values (25 and 23.5 mg/dL) were observed for compounds **1a** (R=H) and **1c** (R=4-F).

#### 2.3.3. Biochemical Parameters of Renal Function

The renal function parameters determined after treatment with the synthesized derivatives are presented in Table 7. Most derivatives produced only limited variations in serum creatinine, indicating the absence of marked alterations in glomerular filtration. Greater variability was observed for urea and uric acid concentrations. Compound **1a** (R=H) exhibited the highest urea and uric acid levels (64.5 mg/dL, respectively 3.78mg/dL), whereas derivatives **1f** (R=2-OCH_3_) and **1j** (R=4-Br) showed the lowest urea concentrations (34.5 and 32.5 mg/dL). In addition, compound **1f** displayed one of the lowest uric acid values (1.8 mg/dL) among the synthesized derivatives, suggesting a more favorable renal biochemical profile.

#### 2.3.4. Evaluation of Hematological Parameters

The blood count is a basic screening test, often representing the first step in establishing the hematological status and diagnosis of various hematological conditions. The hematological parameters determined after treatment with the synthesized derivatives are presented in Table 8. Most erythrocyte indices remained within a relatively narrow range among the experimental groups, indicating the absence of major hematological alterations. However, compound **1e** (R = 4-NO_2_) showed the lowest erythrocyte count (5.35 × 10^6^/μL), hematocrit (33.0%) and MCHC values (28.83 g/dL), together with the highest MCV (62.33 fL). All of these results suggest mild changes in erythrocyte morphology. Leukocyte and platelet counts showed moderate variability among the investigated derivatives without a consistent pattern indicative of hematological toxicity.

### 2.4. Evaluation of Biochemical Parameters in Liver Homogenate

**Figure 3:**
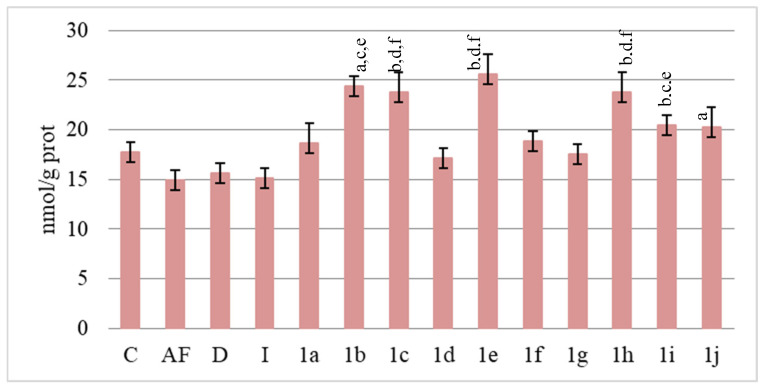
Determination of MDA concentration in liver homogenate samples **1a**–**j**. a: *p* < 0.10 vs. ferulic acid; b: *p* < 0.05 vs. ferulic acid; c: *p* < 0.10 vs. diclofenac; d: *p* < 0.05 vs. diclofenac; e: *p* < 0.10 vs. indomethacin; f: *p* < 0.05 vs. indomethacin.

**Figure 4:**
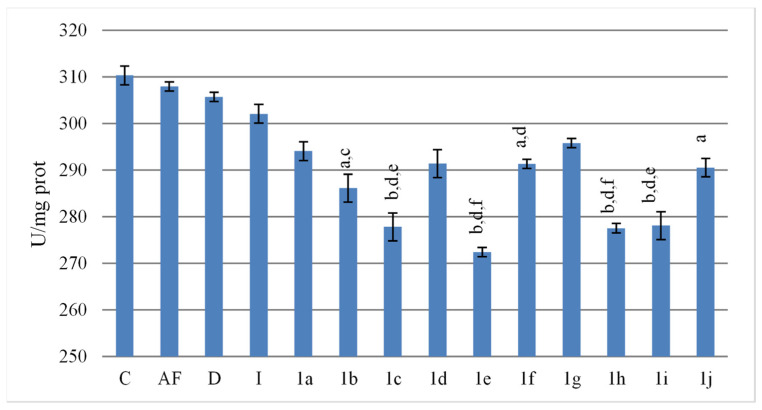
Determination of catalase activity in liver homogenate samples **1a**–**j**. a: *p* < 0.10 vs. ferulic acid; b: *p* < 0.05 vs. ferulic acid; c: *p* < 0.10 vs. diclofenac; d: *p* < 0.05 vs. diclofenac; e: *p* < 0.10 vs. indomethacin; f: *p* < 0.05 vs. indomethacin.

**Figure 5:**
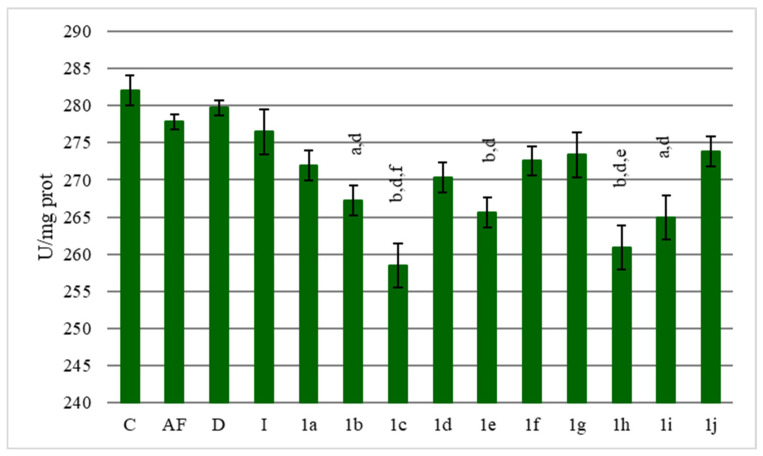
Determination of superoxide dismutase activity in liver homogenate samples. a: *p* < 0.10 vs. ferulic acid; b: *p* < 0.05 vs. ferulic acid; d: *p* < 0.05 vs. diclofenac; e: *p* < 0.10 vs. indomethacin; f: *p* < 0.05 vs. indomethacin.

The oxidative stress biomarkers determined in liver homogenates are summarized in Table 9, whereas the individual changes in malondialdehyde (MDA), catalase (CAT), and superoxide dismutase (SOD) are illustrated in Figure 3, Figure 4 and Figure 5. Collectively, the results demonstrate differences among the synthesized derivatives regarding their ability to modulate oxidative stress.

#### 2.4.1. Determination of Malonaldehyde

The results obtained in determining the MDA concentration in the liver homogenate samples, for the analyzed groups, are presented in Figure 3.

Among the synthesized derivatives, compound **1d** (R = 4-OH, 3-OCH_3_) exhibited the lowest MDA concentration (17.15 nmol/g protein), followed by **1g** (R = 2-OH; 17.56 nmol/g protein), indicating the most limited increase in hepatic lipid peroxidation within the investigated series. Compounds **1a** (R=H) and **1f** (2-OCH_3_) also showed comparatively low MDA levels (18.61 and 18.82 nmol/g protein). In contrast, derivative **1e** (R = 4-NO_2_) produced the highest MDA concentration (25.55 nmol/g protein), while compounds **1b** (R=4-Cl), **1c** (R=4—F), and **1h** (R=2,6-di Cl) also displayed relatively elevated values (24.37, 23.78 respectively 23.80 nmol/g protein). Nevertheless, MDA concentrations for all synthesized derivatives remained higher than those recorded for the control and reference groups.

#### 2.4.2. Determination of Catalase (CAT) and Superoxide Dismutase (SOD) Activity

The activity of CAT is modified by derivatives of ferulic acid, depending on the radical grafted on the unit of ferulic acid and is represented in Figure 4. In hepatic homogenate, the activity of superoxidismutases present in the cytoplasm and mitochondria of hepatocytes was evaluated and represented in Figure 5.

CAT and SOD activities showed a similar response pattern among the investigated derivatives (Figure 4 and Figure 5 and Table 9). The highest CAT activities were observed for compounds **1g** (295.83 U/mg protein, R = 2-OH), **1a** (294.12 U/mg protein, R = H), **1d** (291.42 U/mg protein, R = 4-OH, 3-OCH_3_), **1f** (291.37 U/mg protein, R = 2-OCH_3_), and **1j** (290.55 U/mg protein, R = 4-Br), indicating better preservation of endogenous antioxidant enzyme activity. A similar trend was observed for SOD, with the highest activities recorded for compounds **1j** (273.87 U/mg protein), **1g** (273.39 U/mg protein), **1f** (272.56 U/mg protein), **1a** (271.95 U/mg protein), and **1d** (270.32 U/mg protein). In contrast, compounds **1e** (R = 4-NO_2_), **1c** (R = 4-F), **1h** (R = 2,6-di Cl), and **1i** (R = 4-N(CH_3_)_2_) exhibited the lowest CAT (272.42, 277.86, 277.55, and 278.10 U/mg protein, respectively) and SOD (265.58, 258.46, 260.88, and 264.93 U/mg protein, respectively) activities, suggesting a less favorable modulation of oxidative stress-related biomarkers.

#### 2.4.3. Determination of Transaminase Activity

**Figure 6:**
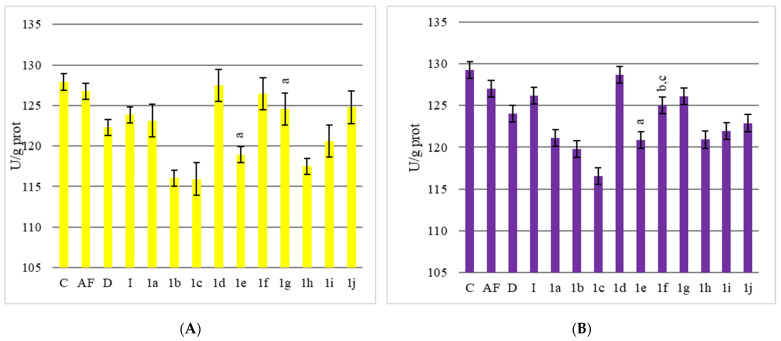
(**A**). Determination of ALT activity in liver homogenate samples treated with compounds **1a**–**j**. a: *p* < 0.10 vs. ferulic acid. (**B**). Determination of AST activity in liver homogenate samples treated with compounds **1a**–**j**. a: *p* < 0.10 vs. ferulic acid; b: *p* < 0.05 vs. ferulic acid; c: *p* < 0.05 vs. indomethacin.

In hepatic homogenate, the transaminase activity was assessed by determining the alanine aminotransferase (ALT) activity and aspartate aminotransferase (AST) activity, represented in Figure 6 as U/g prot. Among the investigated derivatives, compounds **1c** with R=4-F (ALT: 115.92 U/g protein; AST: 116.53 U/g protein) and **1b** with R=4-Cl (ALT: 116.04 U/g protein; AST: 119.77 U/g protein) exhibited the lowest transaminase activities, indicating the most favorable profile with respect to these hepatic biomarkers. Low ALT and AST values were also observed for compounds **1h** (R=2,6-di Cl, ALT:117.50 U/g protein; AST: 120.91 U/g protein) and **1e** (R=4-NO_2,_ ALT: 118.92 U/g protein, AST: 120.89 U/g protein). In contrast, derivatives **1d** (4-OH, 3-OCH_3_), **1f** (R=2-OCH_3)_, **1g** (R=2-OH), and **1j** (R=4-Br) showed the highest ALT (127.46, 126.43, 124.55, respectively 124.79 U/g protein) and AST (128.69, 124.99, 126.11, respectively 122.90 U/g protein) activities among the synthesized compounds, although the differences remained relatively moderate.

Among the investigated compounds, derivatives **1d** (R = 4-OH, 3-OCH_3_), **1g** (R = 2-OH), **1a** (R = H), and **1f** (R = 2-OCH_3_) exhibited the lowest MDA concentrations (17.15–18.83 nmol/g protein). Compounds **1d** (R = 4-OH, 3-OCH_3_), **1g** (R = 2-OH), **1f** (R = 2-OCH_3_), and **1j** (R = 4-Br) also showed comparatively high CAT (290.55–295.83 U/mg protein) and SOD (270.32–273.87 U/mg protein) activities, suggesting a more favorable modulation of oxidative stress-related biomarkers. In contrast, compounds **1b** (R = 4-Cl), **1c** (R = 4-F), **1e** (R = 4-NO_2_), and **1h** (R = 2,6-di Cl) exhibited higher MDA concentrations (23.78–25.55 nmol/g protein), accompanied by lower CAT (272.42–286.17 U/mg protein) and SOD (258.46–267.20 U/mg protein) activities, indicating a less favorable oxidative stress profile. Interestingly, the derivatives exhibiting the most favorable modulation of these biochemical parameters largely overlapped with those showing the strongest anti-inflammatory activity in the carrageenan- and cotton pellet-induced inflammation models. In particular, derivative **1g** (R = 2-OH) exhibited one of the lowest MDA concentrations (17.56 nmol/g protein) together with high CAT (295.83 U/mg protein) and SOD (273.39 U/mg protein) activities. This favorable biochemical profile was accompanied by one of the highest anti-inflammatory effects, suggesting that modulation of oxidative stress-related parameters may contribute, at least in part, to its anti-inflammatory activity.

## 3. Discussion

### 3.1. Determination of Acute Toxicity

Since all chemical substances may pose toxicological risks, safety evaluation is required prior to pharmacological validation. Acute toxicity studies are mandatory under standard regulatory guidelines and constitute a crucial stage in drug discovery and development [37].

The variability of the LD_50_ values suggests that subtle structural modifications of the aromatic ring significantly influence the toxicological behavior of these hybrid molecules. The observed differences in acute toxicity most likely reflect changes in the physicochemical and metabolic properties introduced by the aromatic substituents, which may influence absorption, tissue distribution, biotransformation, and the formation of reactive metabolites. However, these mechanisms were not directly investigated in the present study and therefore remain to be confirmed. These findings are consistent with previous reports demonstrating that structural modifications of the aromatic ring in thiazolidin-4-one derivatives markedly influence both biological activity and toxicity. For example, Vasincu et al. reported that the incorporation of electron-withdrawing substituents, particularly fluorine- and nitro-containing groups, reduced the toxicity of ibuprofen–thiazolidin-4-one hybrids while preserving or even improving their anti-inflammatory activity [38].

The favorable safety profile observed for compounds **1c** (R=4-F) and **1e** (R=4-NO_2_) is also in agreement with the generally low toxicity reported for ferulic acid and its derivatives, which have consistently demonstrated good tolerability in preclinical studies [15]. Although the precise mechanisms responsible for the observed differences remain to be elucidated, the present findings support the hypothesis that the electronic properties and substitution pattern of the aromatic ring contribute significantly to the acute toxicity profile of ferulic acid-derived thiazolidin-4-one hybrids and should therefore be considered in the future optimization of this chemical series.

### 3.2. Determination of In Vivo Anti-Inflammatory Potential

The anti-inflammatory activity of the **1a**–**j** derivatives is likely associated with the complementary biological roles of both pharmacophoric fragments. Ferulic acid primarily acts as a regulator of oxidative stress and inflammation by activating the Nrf2/HO-1 antioxidant pathway, which enhances the expression of endogenous antioxidant enzymes and restores cellular redox homeostasis. In parallel, ferulic acid suppresses the activation of several redox-sensitive inflammatory signaling pathways, including NF-κB, mitogen-activated protein kinase (MAPK), Janus kinase/signal transducer and activator of transcription (JAK/STAT), and the NOD-like receptor family pyrin domain-containing 3 (NLRP3) inflammasome, ultimately reducing the expression of pro-inflammatory cytokines, such as tumor necrosis factor-α (TNF-α), interleukin-1β (IL-1β) and interleukin-6 (IL-6), as well as inflammatory enzymes including cyclooxygenase-2 (COX-2) and inducible nitric oxide synthase (iNOS) [9,32].

In contrast, the thiazolidin-4-one scaffold mainly contributes to the direct modulation of molecular targets involved in the inflammatory cascade. Numerous derivatives belonging to this class have been reported to inhibit COX-2 and 5-lipoxygenase (5-LOX), thereby reducing the biosynthesis of prostaglandins and leukotrienes, while also suppressing iNOS activity and nitric oxide production. Furthermore, several thiazolidin-4-one derivatives interfere with NF-κB activation and exhibit affinity toward peroxisome proliferator-activated receptor gamma (PPARγ), a nuclear receptor involved in the resolution of inflammation and macrophage polarization [33,34]. Consequently, while the ferulic acid moiety predominantly limits the initiation and amplification of oxidative stress-driven inflammatory signaling, the thiazolidin-4-one scaffold mainly targets downstream inflammatory enzymes and mediators. The coexistence of these complementary pharmacophores may therefore explain the pronounced anti-inflammatory activity observed for the synthesized compounds through a multitarget mechanism of action.

These findings indicate that the incorporation of the thiazolidin-4-one scaffold into the ferulic acid structure leads to compounds with enhanced anti-inflammatory potential, supporting their further investigation as potential therapeutic agents.

#### 3.2.1. Acute Inflammation Model Induced with Carrageenan in Rats

The carrageenan-induced hind paw edema model is extensively employed for evaluating the anti-inflammatory potential of novel pharmacologically active compounds, as it reproduces the biphasic inflammatory response associated with the sequential release of inflammatory mediators [39]. The observation that the maximum anti-inflammatory effect was achieved 24 h after administration for all investigated derivatives suggests that the molecular hybridization strategy successfully generated compounds capable of maintaining their pharmacological activity over an extended period. Unlike most of the investigated derivatives, compound **1j** (R=4-Br) did not show an increased inhibitory effect at 24 h. Its inhibition values at 2 h and 24 h were nearly identical (48.10 ± 5.04% and 47.43 ± 6.21%, respectively), and the minor numerical decrease was within the experimental variability. Therefore, this difference was not considered biologically meaningful but rather indicated that the moderate early anti-inflammatory effect of **1j** was not sustained or enhanced during the later phase of the inflammatory response. Moreover, the differences observed among the synthesized derivatives indicate that aromatic substitution substantially modulates the anti-inflammatory response, although no unequivocal relationship between electron-donating and electron-withdrawing substituents could be established. Instead, the biological activity appears to depend on a combination of the electronic properties, steric effects, and the position of the substituent on the aromatic ring. Because the present work was conducted using a single dose and did not include pharmacokinetic investigations, this observation should be interpreted with caution.

These findings are consistent with previous reports demonstrating that structural modifications of the aromatic substituents markedly influence the anti-inflammatory activity of thiazolidin-4-one derivatives. Liaras et al. emphasized that the biological activity of this scaffold is highly dependent on the substitution pattern of the aromatic ring and on its ability to modulate cyclooxygenase (COX) and lipoxygenase (LOX) pathways [33]. Similarly, Mech et al. highlighted that subtle structural modifications of thiazolidin-4-one derivatives may substantially alter their pharmacological profile, confirming the importance of rational aromatic substitution in optimizing anti-inflammatory activity [34].

#### 3.2.2. Anti-Inflammatory Effect on Chronic Inflammation Model

The cotton pellet-induced granuloma model is widely used to evaluate compounds active against chronic inflammation because it simultaneously assesses the transudative and proliferative phases of the inflammatory response [40]. In the present study, the synthesized ferulic acid-derived thiazolidin-4-one derivatives exhibited a more pronounced inhibitory effect on granuloma formation than on the transudative component, suggesting that these compounds may preferentially interfere with cellular events associated with chronic inflammation, including fibroblast proliferation and granulomatous tissue development.

The biological behavior observed in the chronic inflammation model indicates that subtle variations in aromatic substitution substantially influence the pharmacological profile of these hybrids. The experimental findings do not support a simple electronic structure–activity relationship, but rather suggest that multiple structural features collectively determine the anti-inflammatory response. Interestingly, compound **1e**, bearing a para-nitro substituent, exhibited pronounced inhibitory activity in both phases of chronic inflammation, suggesting that this substitution pattern provides a balanced modulation of both the early exudative response and the subsequent proliferative processes involved in granuloma formation. In contrast, several other derivatives displayed preferential activity toward one inflammatory phase, indicating that subtle structural modifications may differentially influence distinct stages of the inflammatory response. The favorable biological profile observed for compound **1e** is therefore in agreement with previous findings [33,34] indicating that para-nitro substitution may enhance the anti-inflammatory activity of selected thiazolidin-4-one derivatives, although this effect appears to depend on the overall molecular framework rather than on the electronic properties of the substituent alone.

### 3.3. Evaluation of Some Biochemical and Hematological Parameters in a Chronic Inflammation Model

#### 3.3.1. Biochemical Parameters for Assessing Liver Function

The hepatic biochemical profile indicates that chronic inflammation was associated with increased activities of AST, ALT, and LDH compared with the healthy control group, confirming the systemic impact of the inflammatory process. Treatment with the synthesized derivatives generally attenuated these alterations, while total and direct bilirubin remained within a relatively narrow range, suggesting that bilirubin metabolism and hepatic excretory function were largely preserved. Collectively, these findings indicate that the investigated compounds produced only limited hepatic alterations under the experimental conditions, despite their pronounced anti-inflammatory activity. The greater variability observed for LDH than for AST and ALT is consistent with the different degrees of oxidative membrane damage induced by the investigated derivatives, which agrees with the MDA, CAT, and SOD findings further discussed.

Since ALT is considered the most sensitive marker of hepatocellular injury, whereas AST is less liver-specific because of its distribution in several extrahepatic tissues, the overall biochemical profile suggests a low degree of hepatocellular damage [41]. Moreover, the absence of marked changes in bilirubin concentrations indicates that hepatic metabolic and excretory functions remained largely preserved despite chronic inflammation, while the moderate LDH variations are consistent with limited cellular injury rather than extensive tissue damage [42]. 

#### 3.3.2. Biochemical Parameters of Lipid Profile

Alterations in lipid metabolism frequently accompany chronic inflammatory conditions as a consequence of cytokine-mediated disturbances in hepatic lipid synthesis, transport, and utilization [43]. Only moderate changes in the lipid profile were observed following administration of the synthesized ferulic acid-derived thiazolidin-4-one derivatives, suggesting that these compounds exerted limited effects on systemic lipid homeostasis despite the inflammatory condition. Although variations in total cholesterol, LDL cholesterol, HDL cholesterol, and triglyceride concentrations were detected among the experimental groups, the overall lipid profile remained comparable to that observed for the reference anti-inflammatory drugs, supporting their favorable systemic safety profile.

The preservation of lipid homeostasis may be associated with the anti-inflammatory activity of the molecular hybrids and their overall redox safety profile, as reflected by the absence of marked alterations in oxidative stress-related biomarkers. Chronic inflammation is known to impair lipid metabolism through sustained production of pro-inflammatory cytokines and reactive oxygen species, which alter hepatic lipid handling and lipoprotein metabolism. Consequently, maintaining a relatively stable lipid profile further supports the ability of these compounds to control inflammation without inducing additional metabolic disturbances [43].

#### 3.3.3. Biochemical Parameters of Renal Function

The renal biochemical profile indicates that the synthesized ferulic acid-derived thiazolidin-4-one derivatives did not produce marked alterations in kidney function during chronic inflammation. Creatinine values remained relatively stable across the experimental groups and were numerically comparable to those observed for the reference anti-inflammatory drugs, suggesting preserved glomerular filtration. Although moderate variations in urea and uric acid concentrations were detected, these changes were not consistently greater than those observed for diclofenac or indomethacin, indicating the absence of substantial renal impairment under the experimental conditions.

Creatinine is considered one of the most reliable indicators of glomerular filtration rate, whereas urea and uric acid provide complementary information regarding renal excretory function and nitrogen metabolism [44]. Consequently, the overall stability of these biochemical parameters suggests that the anti-inflammatory activity of the synthesized derivatives was not accompanied by clinically relevant nephrotoxicity. This observation is particularly important for compounds intended for the long-term management of chronic inflammatory conditions, where preservation of renal function represents a key safety requirement.

#### 3.3.4. Evaluation of Hematological Parameters

The hematological evaluation further supports the favorable systemic tolerability of the synthesized ferulic acid-derived thiazolidin-4-one derivatives. Although slight variations were observed for selected hematological parameters, most values remained within the physiological reference ranges, indicating the absence of clinically relevant hematological toxicity under the experimental conditions. The moderate increases in leukocyte and platelet counts are consistent with the inflammatory response induced by the experimental model rather than with compound-related adverse effects, while erythrocyte indices remained generally stable throughout the experimental groups.

Hematological parameters are sensitive indicators of systemic toxicity and are widely used to evaluate the safety of newly synthesized pharmacologically active compounds [45]. In particular, leukocyte and platelet counts are closely associated with inflammatory responses, whereas erythrocyte indices may provide early evidence of alterations in erythropoiesis or tissue injury. Therefore, the overall stability of the hematological profile observed in the present study further supports the favorable safety profile of the investigated derivatives during chronic inflammation.

When the hepatic, renal, lipid, and hematological findings were considered together, compound **1b** (R = 4-Cl) exhibited the most balanced systemic safety profile among the investigated derivatives. Its favorable hepatic biochemical parameters were accompanied by relatively preserved renal function, moderate lipid alterations, and no major disturbances in erythrocyte-related indices. Compound **1f** (R=2-OCH_3_) also showed a comparatively favorable systemic profile, particularly regarding renal function, although its hepatic and triglyceride values were less advantageous than those observed for **1b** (R=4-Cl).

### 3.4. Evaluation of Biochemical Parameters in Liver Homogenate

#### 3.4.1. Determination of Malonaldehyde

The MDA results indicate that the synthesized derivatives differed substantially in their influence on hepatic lipid peroxidation. The comparatively lower MDA concentrations associated with the hydroxyl-containing derivatives suggest that the availability and position of proton-donating groups may contribute to limiting free-radical-mediated membrane oxidation. In particular, the favorable behavior of the 4-hydroxy-3-methoxy- (**1d**) and 2-hydroxy (**1g**)-substituted derivatives is consistent with the capacity of phenolic hydroxyl groups to stabilize radical intermediates through hydrogen donation and resonance delocalization.

Conversely, the pronounced MDA increase associated with the para-nitro (**1e**) derivative suggests a less favorable redox profile. Nitro groups may undergo enzymatic reduction to reactive intermediates while consuming intracellular reducing equivalents, a process that may contribute to an increased oxidative burden in hepatocytes. However, the elevated MDA values observed for several halogenated derivatives indicate that the redox behavior of this series cannot be attributed exclusively to electron-withdrawing or electron-donating effects. Instead, the combined influence of substituent position, electronic properties, metabolic transformation, and the overall molecular framework probably determines the susceptibility to hepatic lipid peroxidation.

Because MDA represents a terminal product of polyunsaturated fatty-acid peroxidation, its increase reflects enhanced oxidative membrane damage [46]. The relatively limited MDA elevation observed for selected derivatives therefore suggests a more favorable hepatic redox profile, although none of the synthesized compounds reproduced the low values measured for ferulic acid or the control group. These findings should consequently be interpreted together with CAT and SOD activities rather than as independent evidence of antioxidant protection.

#### 3.4.2. Determination of Catalase (CAT) and Superoxide Dismutase (SOD) Activity

CAT and SOD constitute the primary enzymatic defense against oxidative stress; therefore, preservation of their activity reflects the ability of a compound to maintain intracellular redox homeostasis [47]. The overall pattern observed for both enzymes closely paralleled the MDA results, suggesting that the synthesized derivatives modulated oxidative stress through a common mechanism. Derivatives bearing hydroxyl- and methoxy-containing substituents generally preserved CAT and SOD activities more efficiently, whereas compounds containing nitro (**1e**), fluoro (**1c**), dichloro (**1h**), or dimethylamino (**1i**) substituents produced a more pronounced reduction in both antioxidant enzymes.

These findings further support the structure–activity relationship observed for the hepatic oxidative stress biomarkers. Although no single physicochemical property can fully explain the biological behavior of the investigated derivatives, phenolic substituents appear to provide greater protection against oxidative damage, whereas strongly electron-withdrawing substituents may be associated with a higher oxidative burden, possibly reflecting metabolic activation and the subsequent generation of reactive intermediates. Consequently, the activities of the antioxidant enzymes appear to depend on the combined influence of substituent electronics, steric factors, and biotransformation rather than on a single structural feature. 

In our previous study [35], the investigated ferulic acid-derived thiazolidin-4-one derivatives exhibited marked antioxidant activity in chemical assays based on DPPH and ABTS radical scavenging. However, the present in vivo investigation revealed a different oxidative stress profile, characterized by increased MDA levels and reduced CAT and SOD activities for several compounds. Such discrepancies are not unexpected, as chemical assays evaluate only the intrinsic electron- or hydrogen-donating capacity of isolated molecules, whereas the in vivo redox balance is additionally influenced by pharmacokinetic properties, tissue distribution, metabolic transformations, and the formation of reactive intermediates. This phenomenon was particularly evident for the 4-nitro derivative (**1e**), which showed good radical-scavenging activity in vitro but produced the highest hepatic MDA levels. Aromatic nitro groups are known to undergo enzymatic nitroreduction, yielding nitroso and hydroxylamine intermediates capable of redox cycling and ROS generation. Therefore, the oxidative burden potentially associated with nitro group metabolism may offset the intrinsic antioxidant potential of the parent molecule and may contribute to increased lipid peroxidation, despite its favorable in vitro radical-scavenging activity [48].

In contrast, compound **1d**, bearing a 4-hydroxy-3-methoxy substituent, exhibited one of the highest radical-scavenging activities in vitro (DPPH radical scavenging ability *EC*_50_ = 9.79 µg/mL; ABTS radical scavenging ability, *EC*_50_ = 1.99 µg/mL) and induced only moderate alterations in hepatic oxidative stress biomarkers, suggesting that phenolic substituents may contribute to a more favorable modulation of redox-related parameters in biological systems than nitro-containing analogs. These findings are consistent with the structure–activity relationship observed in our previous in vitro study, where derivatives bearing phenolic substituents displayed superior radical-scavenging activity compared with nitro-containing (DPPH radical scavenging *EC*_50_ = 24.56 µg/mL; ABTS radical scavenging ability, *EC*_50_ *=* 6.62 µg/mL) ability analogs.

Overall, these findings indicate that the antioxidant activity observed in chemical in vitro assays cannot be directly extrapolated to biological systems, where metabolic activation, biotransformation, tissue distribution, and pharmacokinetic factors substantially influence the in vivo redox status.

#### 3.4.3. Determination of Transaminase Activity

The transaminase profile generally paralleled the oxidative stress biomarkers, suggesting a close relationship between hepatic redox status and intracellular enzyme activity. Derivatives associated with the most pronounced oxidative stress, particularly those producing higher MDA levels together with lower CAT and SOD activities, also tended to exhibit lower ALT and AST activities. This pattern may reflect alterations in hepatocyte membrane integrity and enzyme release associated with oxidative damage rather than direct inhibition of transaminase activity.

The influence of aromatic substitution was again evident, although no single structural feature fully explained the observed biological response. Nitro- and halogen-containing derivatives generally showed a less favorable oxidative profile than hydroxyl-containing analogs, supporting the hypothesis that metabolic activation of strongly electron-withdrawing substituents may contribute to increased oxidative burden. By contrast, derivatives containing hydroxyl and methoxy substituents (**1d**) maintained a more balanced redox profile, which is consistent with the protective effect of phenolic groups against oxidative membrane damage.

Previous studies have shown that ferulic acid itself exerts hepatoprotective effects and may attenuate drug-induced liver injury through its antioxidant activity [49]. The present findings suggest that molecular hybridization with the thiazolidin-4-one scaffold generally preserves this favorable hepatic profile, although the extent of protection remains strongly dependent on the nature of the aromatic substituent.

When all biological endpoints are considered together, compound **1g** (R = 2-OH) emerges as the most promising derivative within the investigated series. It combined one of the strongest anti-inflammatory activities in the carrageenan-induced paw edema model (97.43% inhibition at 24 h) with one of the lowest MDA concentrations (17.56 nmol/g protein) and comparatively high CAT (295.83 U/mg protein) and SOD (273.39 U/mg protein) activities. Although compound **1e** (R = 4-NO_2_) exhibited the highest inhibitory activity in the chronic inflammation model (% inhibition of granulation tissue formation 79.85%), and compound **1b** (R = 4-Cl) showed the most favorable systemic biochemical safety profile, neither displayed the same combination of anti-inflammatory efficacy and favorable modulation of oxidative stress-related biomarkers observed for **1g**. This integrated pharmacological profile supports the selection of compound **1g** as the lead candidate for further optimization and mechanistic investigations.

#### 3.4.4. Study Limitations

The present study represents an initial in vivo pharmacological evaluation of ferulic acid-derived thiazolidin-4-one hybrids. Although preliminary structure–activity relationships were inferred from the observed differences among substituents, these interpretations should be considered exploratory because no molecular docking, QSAR modeling, physicochemical correlation analyses (e.g., Hammett constants or logP), or other computational chemistry approaches were performed. Consequently, the proposed SAR observations require confirmation through complementary computational and mechanistic studies. Furthermore, the molecular mechanisms underlying the anti-inflammatory activity of the investigated derivatives were not directly investigated, and no pharmacokinetic studies were conducted. Future studies addressing molecular targets, pharmacokinetic behavior, and chronic safety will be important to further characterize the therapeutic potential of these compounds.

Another limitation of the present study is that the investigated derivatives were administered at one-tenth of their individual LD_50_ values, whereas diclofenac and indomethacin were administered at fixed pharmacologically established doses. Consequently, although the anti-inflammatory activities of the investigated compounds could be compared qualitatively with those of the reference drugs under the selected experimental conditions, these findings should not be interpreted as evidence of equivalent pharmacological potency or therapeutic superiority. Future dose–response studies, including ED_50_ determination and pharmacokinetic evaluation, will be necessary to establish the relative pharmacological profiles of these compounds.

In addition, animals were allocated to the experimental groups according to body weight rather than by formal randomization. Although this approach minimized baseline differences among groups, it may have introduced a potential source of allocation bias. Furthermore, only male animals were included in the present study, and no histopathological analyses were performed to complement the biochemical findings. Future studies should therefore include both sexes, implement randomized allocation procedures, and integrate histopathological evaluation to provide a more comprehensive assessment of the pharmacological and safety profiles of the investigated derivatives.

## 4. Materials and Methods

Mice and rats are widely used in preclinical pharmacological studies owing to their well-characterized physiology and inflammatory responses, which share important similarities with those observed in humans. Acute toxicity studies in mice provide essential information on the safety profile of different compounds and support dose selection for subsequent pharmacological investigations.

All animal experiments were conducted in accordance with the national legislation governing the use of laboratory animals and with Directive 2010/63/EU of the European Parliament and of the Council on the protection of animals used for scientific purposes. The experimental protocols were reviewed and approved by the Research Ethics Committee of “Grigore T. Popa” University of Medicine and Pharmacy, Iași, Romania (approval dated 7 January 2014). Animals were obtained from the accredited laboratory animal facility of the National Institute for Research and Development in Microbiology and Immunology “Cantacuzino” (Bucharest, Romania) and were clinically healthy at the beginning of the experiment. Diclofenac sodium and indomethacin were selected as positive reference drugs because they are well-established non-steroidal anti-inflammatory drugs (NSAIDs) widely used as standard comparators in experimental models of acute and chronic inflammation, owing to their well-characterized anti-inflammatory efficacy. Tween 80 was used as the vehicle for the preparation of ferulic acid and the investigated compounds. Therefore, animals in the control group received the corresponding volume of Tween 80 alone to exclude any potential effects of the vehicle on the evaluated biological parameters. No prior protocol, including the research question, study design, or statistical analysis plan, was formally registered before the initiation of the study. The experimental design and analysis were defined prior to data collection in accordance with standard laboratory practice.

All efforts were made to minimize animal suffering and to reduce pain and distress throughout the experimental procedures. Animals were acclimatized prior to experimentation and maintained under standard laboratory conditions with free access to food and water. All procedures were performed by trained personnel, and handling was carried out in a manner designed to minimize stress. Where applicable, anesthetic or supportive measures were used in accordance with approved protocols to reduce procedural discomfort.

Animals were monitored regularly for signs of adverse effects, including changes in behavior, locomotor activity, grooming, posture, and food and water intake. No unexpected severe adverse events were observed during the study period, and all procedures were well tolerated within the expected range for the experimental models used.

Humane endpoints were predefined according to institutional ethical guidelines and included severe lethargy, persistent pain-related behavior, marked weight loss, or any signs of systemic distress. Animals were observed at least daily, and more frequently during critical phases of the experiments. No animals reached humane endpoint criteria requiring early euthanasia during the study.

### 4.1. Determination of Acute Toxicity

Acute toxicity testing is a fundamental method for assessing the overall toxic potential of a substance, estimating its median lethal dose (LD_50_). The LD_50_ represents the dose required to cause death in 50% of the exposed test population when administered via a specified route of exposure [50,51]. The acute toxicity study was performed to evaluate the safety profile of the tested compound and to determine the median lethal dose (LD_50_), providing information necessary for dose selection in subsequent pharmacological studies. The toxicological screening was performed on male, white Swiss mice weighing in the range of 17–29 g. They were housed in clean polyethylene cages (one cage for each batch) and acclimatized for 7 days before the start of the experiment, having access to food and water ad libitum throughout this period. The laboratory environmental conditions were relatively constant throughout the experiment, the temperature being 23 ± 2 °C, with a 12 h light and 12 h dark cycle and relative humidity of 40–70%. Before the start of the experiment, the mice were deprived of food for 24 h, receiving only water ad libitum.

Acute toxicity was evaluated by means of the lethal dose 50 test, which consists of administering compounds in doses that increase in geometric progression to calculate the dose at which 50% of the mice included in the experiment die. Eight experimental groups corresponding to increasing dose levels (500, 1000, 2000, 3000, 4000, 5000, 6000, and 6500 mg/kg body weight) were included in the acute oral toxicity study. Each group consisted of eight animals (*n* = 8), resulting in a total of 64 animals used in the experiment. Animals received a single oral administration of the test compound formulated as an emulsion in Tween 80, and survival was monitored at 24, 48, and 72 h, and on days 7 and 14 post-administration. The sample size was determined based on conventional LD_50_ experimental designs reported in the toxicological literature, in which approximately 8–10 rodents per dose group are commonly used to obtain reliable mortality data and permit estimation of the median lethal dose. Therefore, eight animals were allocated to each dose group, which was considered sufficient to characterize the dose–response relationship while avoiding unnecessary animal use. No formal a priori power analysis was performed. No predefined exclusion criteria were established prior to the experiment. All healthy animals allocated to the study were included in the final analysis. No animals or data points were excluded from the analysis.

The investigator responsible for animal allocation and dose administration was aware of group assignment because each experimental group received a different dose level. Survival monitoring and mortality recording were based on objective criteria. No blinding was implemented during the conduct of the experiment or outcome assessment. The outcome measures assessed included mortality rate, clinical signs of toxicity, and survival at 24, 48, and 72 h, and on days 7 and 14 after oral administration. Data analysis and LD_50_ calculation were performed using coded datasets.

To establish the LD_50_, the Spearman-Karber arithmetic method was used, which is based on the following calculation formula [52]:

$$LD_{50}=\;{LD}_{100}-\;\frac{\sum (a+b)}{n}$$

where

a = the difference between two successive doses of the substance administered;

b = the average number of dead animals from two successive batches;

*n* = the number of animals in a batch;

LD_100_ = lethal dose 100 (representing the amount of substance that causes the death of all animals in the experimental group).

### 4.2. In Vivo Anti-Inflammatory Potential

Based on the results obtained in the evaluation of the anti-inflammatory effect by in vitro methods and the evaluation of the degree of toxicity, the 2-(R-phenyl)-3-[3-(4-hydroxy-3-methoxyphenyl)acrylamido]-thiazolidin-4-one derivatives (**4a**–**j**), obtained by structural modulation of ferulic acid, were included in a pharmacological screening that aimed to determine the anti-inflammatory effect in vivo:

- On a model of acute inflammation induced with carrageenan in rats;
- On a model of chronic inflammation induced in rats-the granuloma test.

The carrageenan-induced paw edema [53] and cotton pellet-induced granuloma [54] models are established and validated experimental models that reproduce key features of acute and chronic inflammation, respectively, allowing the assessment of anti-inflammatory activity and facilitating the translation of preclinical findings to inflammatory conditions in humans. The pharmacological dose selected for each synthesized derivative corresponded to one-tenth (1/10) of its experimentally determined LD_50_ value. This dose was selected because the use of one-tenth of the LD_50_ is a widely accepted strategy in preclinical pharmacological studies, allowing the evaluation of biological activity at a dose well below the acute toxic threshold while minimizing the risk of treatment-related toxicity during repeated administration [55].

The sample size was selected based on previous studies employing inflammation models and on common practice in experimental pharmacology, where groups of six rats are generally considered adequate to detect biologically relevant differences while ensuring compliance with the principles of Reduction, Replacement and Refinement (3Rs). No formal a priori sample size calculation was performed. No predefined exclusion criteria were established prior to the experiment. All healthy animals meeting the predefined body weight criteria were included in the study. Data analysis was performed using all collected measurements, and no outlier-based exclusion was applied. No formal randomization procedure was used. Animals were allocated to the dose groups according to body weight to ensure comparable baseline characteristics among groups.

*Data analysis*—Data are presented as mean ± SD (*n* = 6). Statistical analyses were performed using two-way repeated-measures ANOVA followed by Tukey’s multiple-comparison test for the carrageenan-induced acute inflammation model and one-way ANOVA followed by Tukey’s multiple-comparison test for the cotton pellet-induced chronic inflammation model (GraphPad Prism version 11.0.0, GraphPad Software, Boston, MA, USA). Following ANOVA, Tukey’s multiple-comparison test was used to perform all pairwise comparisons among the experimental groups, including comparisons between the synthesized derivatives, the vehicle (Tween 80) control group, and the reference drugs. However, because the primary objective of both inflammation models was to evaluate the anti-inflammatory activity of the synthesized derivatives, only comparisons with the vehicle (Tween 80) control group are reported in the figures and tables. Differences were considered statistically significant at *p* < 0.05. Effect sizes and confidence intervals were not estimated. 

#### 4.2.1. Carrageenan-Induced Acute Inflammation Model in Rats

The carrageenan-induced paw edema model was used as a validated and reproducible model of acute inflammation to assess the anti-inflammatory activity of the tested compound through the measurement of edema formation. The study of the anti-inflammatory effect on the acute inflammation model was carried out on white Wistar rats, adult males, weighing between 140 and 220 g, raised under identical laboratory conditions and acclimatized for 7 days before the start of the experiment, under relatively constant environmental conditions: temperature 23 ± 20 °C, humidity 40–60%, 12 h light/dark cycle. The animals were housed individually in standard polypropylene cages and 18 h before the beginning of the experiment, they were deprived of food, receiving only water ad libitum.

Acute inflammatory edema was induced with carrageenan, according to the working protocols described in the specialized literature with some modifications [39,50,56]. After weighing, the rats were divided into groups of 6 rats (*n* = 6) according to body weight, using a total of 84 rats. The tested compounds (**4a**–**j**) were administered by gavage in doses of 1/10 of the LD_50_ by dispersion in Tween 80. The study also included a group considered a positive control, treated with ferulic acid, two reference groups, treated with diclofenac sodium (5 mg/kg body weight) and indomethacin (1.5 mg/kg body weight) and a control group in which the animals received only the vehicle used for the administration of the study substances, Tween 80 (0.5 mL/100 g body weight). The structure of the groups and the dose administered corresponding to each test compound are presented in Table 10.

According to the working protocol described in the literature, before the administration of the compounds, the volume of the left hind paw of the rat was determined using the LE 7500 digital plethysmometer (Panlab, Cornellà de Llobregat, Spain).

Edema was induced by intraplantar injection, in the left hind paw, of 0.2 mL of 1% k-carrageenan suspension (Sigma Aldrich, Steinheim, Germany) in physiological saline (sodium chloride 0.9 mg/mL). Immediately after the injection of the carrageenan, the compounds to be studied were administered by gavage, after which the volume of the left hind paw was measured 2 h, 4 h, 6 h and 24 h after administration [53,56].

Group allocation and treatment administration were performed by one investigator, whereas paw volume measurements and data analysis were carried out by a second investigator blinded to treatment allocation. The outcome measures included paw edema volume measured at predetermined time intervals following carrageenan administration and the percentage inhibition of edema relative to the control group.

To interpret the results, for each studied group, the difference between the hind paw volume of each rat at different time intervals (2 h, 4 h, 6 h and 24 h) and the initially determined volume was calculated, according to the calculation formula:

∆Vt/c = Vt/c − Vi

where

∆Vt/c = edema volume in the treated/control group;

Vt/c = volume of the left hind paw of the rats in the treated/control group;

Vi = volume of the left hind paw of the rats before administration of carrageenan.

The results were compared with those of the group treated with Tween 80, considered the control group.

To study the anti-inflammatory effect, the percentage of inhibition of acute edema induced with carrageenan was calculated for each time interval (2 h, 4 h, 6 h, 24 h), according to the following calculation formula:

$$\%\;\mathrm{edema}\;\mathrm{inhibition}\;=\;\frac{\left(∆V_{c}-{∆V}_{t}\right)}{{∆V}_{c}}$$

where:

∆V_c_ = edema volume in the control group;

∆V_t_ = edema volume in the treated group.

#### 4.2.2. Chronic Inflammation Model, Induced in Rats-Granuloma Test

The study of the anti-inflammatory effect of the synthesized compounds, derivatives of 2-(R-phenyl)-3-[3-(4-hydroxy-3-methoxyphenyl)acrylamido]-thiazolidin-4-one (**1a**–**j**), on a chronic inflammation model was carried out using the granuloma test, performed as described in the literature with minor modifications [50,57]. This method is widely used for the assessment of chronic inflammation. The cotton pellet-induced granuloma model was used as a validated model of chronic inflammation to assess the effects of the tested compound on exudate formation (transudative phase) and granuloma tissue development (proliferative phase), determined by the wet and dry weights of the cotton pellets, respectively. We worked on a total of 84 animals, divided in groups of 6 rats (*n* = 6), Wistar breed, white, weighing between 140 and 220 g, raised under identical laboratory conditions and acclimatized for 7 days before the start of the experiment, under relatively constant environmental conditions: temperature 23 ± 2 °C, humidity 40–60%, 12 h light/dark cycle and housed individually. 18 h before the start of the experiment, the animals were deprived of food, with water being maintained ad libitum.

The rats were anesthetized with ketamine (80–100 mg/kg body weight), after which they were incised in the interscapulohumeral area, into which two sterile cotton fiber pellets weighing 60 mg were inserted. The pellets were kept in the respective area for 7 days, during which the experimental animals received, at 24 h intervals, by gavage, the tested substances (**1a**–**j**), at a dose of 1/10 of the LD_50_ by dispersion in Tween 80.

The study also included a group considered positive control, treated with ferulic acid, two reference groups, treated with diclofenac sodium (5 mg/kg body weight) and indomethacin (1.5 mg/kg body weight) and a control group in which the animals received only the vehicle used to administer the substances to be studied, namely Tween 80 (0.5 mL/100 g body weight). 

After 7 days, the pellets were extracted, weighed in a wet state, then dried for 18 h at a temperature of 57 °C and weighed again. Outcome measures included wet granuloma weight, dry granuloma weight, and percentage inhibition of granuloma formation. The lower the mass of the pellet, the more intense the anti-inflammatory action of the studied compound is considered.

Group allocation, cotton pellet implantation, and treatment administration were performed by one investigator who was aware of the treatment groups. Outcome assessment, including granuloma excision, drying, and weighing, was performed by a second investigator blinded to group allocation. Animals and tissue samples were identified using coded labels to ensure that the investigator responsible for measurements remained unaware of treatment assignment, in order to minimize potential bias.

### 4.3. Evaluation of Some Biochemical and Hematological Parameters in a Chronic Inflammation Model

The compounds included in the study of the anti-inflammatory effect in a chronic inflammation model, namely the compounds (**1a**–**j**), were further studied regarding the effect on the following parameters:

- Biochemical–alanine aminotransferase (ALT), aspartate aminotransferase (AST), total bilirubin, direct bilirubin, lactate dehydrogenase (LDH), urea, creatinine, uric acid, total cholesterol, LDL cholesterol, HDL cholesterol, triglycerides;
- Hematological–complete blood count.

The experiment was performed on white Wistar rats, adult males, weighing between 140 and 220 g, raised under identical laboratory conditions and acclimatized for 7 days before the start of the experiment, in relatively constant environmental conditions: temperature 23 ± 2 °C, humidity 40–60%, 12 h light/dark cycle. The animals were housed individually in standard polypropylene cages, and 18 h before the start of the experiment, the animals were deprived of food, with water being maintained ad libitum.

At the end of the experiment that followed the effect of the compounds on inflammatory edema induced by the granuloma test, the rats were anesthetized by administering ketamine i.p. 80 mg/kg body weight. After immobilization by fixing the limbs, the thorax was opened together with part of the abdominal area, and blood was collected by cardiac puncture for the determination of biological and hematological parameters and organs.

The study also included a second control group (C2 healthy), which included healthy rats that were not induced to develop chronic inflammation by the granuloma test, and which received Tween 80, 0.5 mL/100 g body weight, for 7 days.

Part of the collected blood was introduced into vacutainers with EDTA (to avoid coagulation); these samples were used for hematological determinations. For these determinations, the ABC VET analyzer was used. For serological determinations, after coagulation, the blood samples were centrifuged for 14 min at 3500 rpm. Biochemical parameters determined were: AST, ALT, total bilirubin, direct bilirubin, LDH, urea, creatinine, uric acid, total cholesterol, LDL cholesterol, HDL cholesterol, triglycerides. For biochemical determinations, the RX Imola analyzer series 7201-0505 produced by “RANDOX LABORATORIES Ltd.” (London, UK) and “FURUNO ELECTRIC Co., Ltd.” (Nishinomiya, Japan) was used.

### 4.4. Evaluation of Biochemical Parameters in Liver Homogenate

#### 4.4.1. Preparation of Liver Homogenate

Liver samples were rapidly excised, washed with ice-cold saline, and homogenized (1:10, *w*/*v*) in phosphate buffer (50 mM, pH 7.4) using a mechanical homogenizer (SilentCrusher M, Heidolph Instruments, Schwabach, Germany) at 4 °C. The homogenates were centrifuged at 10,000× *g* for 15 min at 4 °C, and the supernatants were collected for further biochemical analyses.

#### 4.4.2. Determination of Malondialdehyde (MDA)

Malondialdehyde (MDA) levels were determined using the thiobarbituric acid (TBA) assay. MDA reacts with TBA to form a pink chromogen with maximum absorbance at 532 nm. Briefly, 200 µL of liver homogenate was mixed with 600 µL of TBA solution in glacial acetic acid and heated at 95 °C for 60 min. After cooling in an ice bath, the samples were kept at room temperature for 10 min, and absorbance was measured at 532 nm against a blank. MDA concentration was calculated using a calibration curve prepared with standard MDA solutions (Sigma-Aldrich Lipid Peroxidation Assay Kit).

#### 4.4.3. Determination of Catalase Activity (CAT)

Catalase activity was determined according to the method described by Aebi, based on the decomposition of hydrogen peroxide, monitored as a decrease in absorbance at 240 nm. A volume of 20 µL of sample was added to 3 mL of hydrogen peroxide solution (10.5 mM) prepared in phosphate buffer (50 mM, pH 7). The decrease in absorbance was recorded at 240 nm against a blank containing phosphate buffer. The time required for the absorbance to decrease from 0.45 to 0.40 was used to calculate catalase activity, expressed as U/mg protein (Aebi, 1984) [58].

#### 4.4.4. Determination of Superoxide Dismutase Activity (SOD)

Superoxide dismutase activity was determined using a RANSOD assay kit (Randox Laboratories), based on the inhibition of the reduction of 2-(4-iodophenyl)-3-(4-nitrophenol)-5-phenyltetrazolium chloride (INT) by superoxide radicals generated by xanthine oxidase.

Briefly, 15 µL of diluted liver homogenate (1:25) was mixed with 500 µL of substrate solution containing xanthine (0.05 mmol/L) and INT (0.025 mmol/L) in phosphate buffer. After mixing, 75 µL of xanthine oxidase (80 U/L) was added. Absorbance was measured at 505 nm after 30 s and 3 min. SOD activity was expressed as U/mg protein.

#### 4.4.5. Determination of Transaminase Activity-Alanine Aminotransferase Activity (ALT) and Aspartate Aminotransferase Activity (AST)

ALT activity was determined using a commercial assay kit (Randox), based on the conversion of L-alanine and α-ketoglutarate to pyruvate, followed by oxidation of NADH to NAD^+^, monitored at 340 nm. Briefly, 0.2 mL of sample was mixed with 2 mL of reaction mixture containing L-alanine, lactate dehydrogenase, α-ketoglutarate, and NADH in Tris buffer. Absorbance was recorded at 340 nm after 1 min and subsequently at 1, 2, and 3 min. The change in absorbance per minute was used to calculate ALT activity, expressed as U/g protein.

AST activity was determined using a commercial assay kit (Randox), based on the conversion of L-aspartate and α-ketoglutarate to oxaloacetate, followed by NADH oxidation, monitored at 340 nm. A volume of 0.2 mL of sample was mixed with 2 mL of reaction mixture containing L-aspartate, malate dehydrogenase, α-ketoglutarate, and NADH in Tris buffer. Absorbance was measured at 340 nm after 1 min and at 1, 2, and 3 min. The change in absorbance per minute was used to calculate AST activity, expressed as U/g protein.

#### 4.4.6. Reagents and Equipment

All reagents were of analytical grade. Thiobarbituric acid, glacial acetic acid, hydrogen peroxide, and buffer components were purchased from standard suppliers. Commercial assay kits for SOD, ALT, and AST were obtained from Randox Laboratories (London, UK). Spectrophotometric measurements were performed using a UV–Vis spectrophotometer (Jasco V-550, Kingsgrove, Australia), and incubation steps were carried out in a thermostatic water bath (Memmert, Schwabach, Germany).

*Data analysis:* Data are presented as mean ± SEM (*n* = 6) for serum biochemical, renal, lipid profile, and hematological parameters, and as mean ± SD (*n* = 6) for hepatic oxidative stress biomarkers (MDA, CAT, and SOD). Statistical analyses were performed using one-way ANOVA followed by Tukey’s multiple-comparison test (GraphPad Prism version 11.0.0, GraphPad Software, Boston, MA, USA). Following ANOVA, Tukey’s multiple-comparison test was used to perform all pairwise comparisons among the experimental groups. However, the comparisons reported in the manuscript were selected according to the biological objective of each analysis. For serum biochemical, renal, lipid profile, and hematological parameters, comparisons with the healthy and vehicle (Tween 80) control groups are reported to evaluate the systemic safety profile of the synthesized derivatives. No statistically significant differences were observed between the healthy control and vehicle control groups (*p* > 0.05). For hepatic oxidative stress biomarkers (MDA, CAT, and SOD), comparisons with the reference compounds (ferulic acid, diclofenac, and indomethacin) are reported to assess the relative effects of the synthesized derivatives under the experimental conditions. Differences were considered statistically significant at *p* < 0.05. 

## 5. Conclusions

The present study demonstrated that the synthesized ferulic acid-derived thiazolidin-4-one derivatives exhibit distinct biological profiles depending on the nature of the aromatic substituent. Acute toxicity studies indicated moderate toxicity for the investigated series, while the para-fluoro (**1c**) and para-nitro (**1e**) derivatives displayed the highest LD_50_ values (4812.5 mg/kg body, respectively 5000 mg/kg body).

Among the investigated compounds, derivative **1g** (R = 2-OH) emerged as the most promising overall candidate, combining the strongest anti-inflammatory activity in the acute inflammation model (% inhibition of inflammatory edema at 24 h 97.43%) of with low MDA levels (17.56 nm/g ptotein) and comparatively preserved activities of the antioxidant enzymes CAT (295.83 U/mg protein) and SOD (273.39 U/mg protein). In contrast, compound **1e** (R = 4-NO_2_) exhibited the highest inhibitory effect on granuloma formation in the chronic inflammation model (% inhibition of granulation tissue 79.85%), although it was associated with less favorable oxidative stress-related biomarker values than the hydroxyl-substituted derivatives. Derivative **1b** (R = 4-Cl) showed the most favorable hepatic biochemical profile, whereas compound **1d** (R = 4-OH, 3-OCH_3_) consistently demonstrated favorable modulation of oxidative stress-related biomarkers (MDA: 17.15 nmol/g protein; CAT: 291.42 U/mg protein and SOD: 270.32 U/mg protein). Overall, several of the investigated derivatives demonstrated favorable anti-inflammatory profiles under the experimental conditions employed.

However, because the synthesized compounds and the reference drugs were administered using different dosing regimens, direct comparisons of their anti-inflammatory effects should be interpreted with caution.

Collectively, these findings indicate that hydroxyl-containing substituents, particularly the ortho-hydroxyl (**1g**) and para hydroxy -meta methoxy (**1d**) substitution patterns, provide the most balanced combination of anti-inflammatory efficacy and preservation of hepatic redox homeostasis, whereas strongly electron-withdrawing substituents, especially the nitro group, although capable of producing marked anti-inflammatory effects, were associated with a less favorable oxidative stress profile.

Overall, the present findings identify **1g** (R=2-OH) as the lead compound within the investigated series, while compounds **1d** (R=4-OH, 3-OCH_3_) and **1b** (R=4-Cl) also demonstrated favorable biological profiles because of their favorable modulation of oxidative stress-related biomarkers and hepatic biochemical profile, respectively. These results support the continued preclinical investigation of selected ferulic acid-derived thiazolidin-4-one derivatives as multifunctional anti-inflammatory agents with a favorable redox safety profile.

In addition, comparisons with the reference drugs should be interpreted cautiously because the synthesized derivatives were administered at one-tenth of their individual LD_50_ values, whereas diclofenac and indomethacin were administered at fixed pharmacologically established doses. Consequently, the present data do not allow direct conclusions regarding the relative pharmacological potency or therapeutic equivalence of the investigated compounds compared with the reference drugs. 

## Figures and Tables

**Table 1:** Lethal dose 50 (LD_50_ in mg/kg body) for thiazolidin-4-one derivatives **1a**–**j**.

Compound	LD_50_ (mg/kg Body)	Compound	LD_50_ (mg/kg Body)
**1a**	987.5	**1f**	1350
**1b**	1590	**1g**	1250
**1c**	4812.5	**1h**	1550
**1d**	925	**1i**	1750
**1e**	5000	**1j**	1490
**Ferulic acid**	2875		

**Table 2:** Anti-inflammatory effect (% inhibition of inflammatory edema) of the tested compounds (**1a**–**j**, **G1**–**10**) compared to ferulic acid (G11), diclofenac sodium (G12) and indomethacin (G13) at different time intervals.

Group/Compound	% Inhibition of Inflammatory Edema			
	2 h	4 h	6 h	24 h
**G1/1a**	18.98 ± 2.6	17.85 ± 5.14	20.03 ± 2.45	87.17 ± 6.33
**G2/1b**	34.17 ± 4.87	27.38 ± 3.06	22.35 ± 2.26	73.07 ± 5.86 *
**G3/1c**	31.64 ± 6.49 *	30.35 ± 4.28	32.94 ± 3.01	73.07 ± 4.87
**G4/1d**	27.84 ± 8.54	2.38 ± 1.98	23.52 ± 5.50	91.02 ± 9.12
**G5/1e**	56.96 ± 5.35	38.09 ± 9.82	22.35 ± 4.81 *	76.92 ± 2.56 **
**G6/1f**	34.68 ± 2.94	31.42 ± 3.21	32.23 ± 2.77	95.38 ± 8.70
**G7/1g**	55.69 ± 2.21	32.14 ± 1.61	47.05 ± 2.16	97.43 ± 7.09 **
**G8/1h**	56.32 ± 2.31	50.01 ± 8.44 *	50.58 ± 5.61	78.84 ± 4.33
**G9/1i**	53.16 ± 4.09	46.42 ± 3.10	57.64 ± 3.79	94.87 ± 6.61 **
**G10/1j**	48.10 ± 5.04	34.52 ± 7.95	57.64 ± 4.07 *	47.43 ± 6.21
**G11/Ferulic acid**	51.89 ± 2.20	47.61 ± 2.41	76.51 ± 9.27	92.30 ± 8.25
**G12/Diclofenac sodium**	54.43 ± 3.48	53.57 ± 2.43	43.52 ± 2.25	94.87 ± 11.61
**G13/Indomethacin**	73.41 ± 2.70	64.28 ± 1.81	75.29 ± 2.15	96.15 ± 11.10

The vehicle-treated control group received Tween 80 and was used for calculating the percentage inhibition of paw edema; therefore, its inhibition value was 0% at all time points and was not included in the table. Data are presented as mean ± SD (*n* = 6). * *p* < 0.05 and ** *p* < 0.01 compared with the vehicle (Tween 80) control group.

**Table 3:** Effect of 2-(R-phenyl)-3-[3-(4-hydroxy-3-methoxyphenyl) acrylamido]-thiazolidin-4-one compounds (**1a**–**j**) on the transudative process in chronic inflammatory edema induced in rats.

Group/Compound	Administered Dose (mg/kg Body Weight/Day)	Mean Weight of Wet Pellet (mg)	% Inhibition
**G1 (1a)**	98.75	0.771 ± 0.19 *	42.47
**G2 (1b)**	159.00	1.155 ± 0.28	13.81
**G3 (1c)**	481.25	0.786 ± 0.06 *	41.35
**G4 (1d)**	92.50	0.887 ± 0.14	33.81
**G5 (1e)**	500.00	0.834 ± 0.07 *	37.77
**G6 (1f)**	135.00	1.104 ± 0.15 *	17.62
**G7 (1g)**	125.00	0.806 ± 0.09	39.86
**G8 (1h)**	155.00	0.968 ± 0.13	27.77
**G9 (1i)**	175.00	0.896 ± 0.18	33.14
**G10 (1j)**	149.00	1.091 ± 0.16	18.59
**G11 (Ferulic acid)**	287.50	0.853 ± 0.14	36.35
**G12 (Diclofenac sodium)**	5.00	0.768 ± 0.08	42.69
**G 13 (Indomethacin)**	1.50	0.878 ± 0.11	34.48
**G14 (Tween 80)**	0.5 mL/100 g	1.34 ± 0.19	-

Data are presented as mean ± SD (*n* = 6). * *p* < 0.05 compared with the vehicle (Tween 80) control group.

**Table 4:** Effect of 2-(R-phenyl)-3-[3-(4-hydroxy-3-methoxyphenyl)acrylamido]-thiazolidin-4-one compounds (**1a**–**j**) on the proliferative process (granulation tissue formation) in chronic inflammatory edema induced in rats.

Group/Compound	Administered Dose (mg/kg Body Weight/Day)	Mean Weight of Dry Pellets (mg)	% Inhibition
**G1 (1a)**	98.75	0.617 ± 0.03 *	21.31
**G2 (1b)**	159.00	0.768 ± 0.05 *	2.05
**G3 (1c)**	481.25	0.227 ± 0.12	71.05
**G4 (1d)**	92.50	0.209 ± 0.178 *	73.35
**G5 (1e)**	500.00	0.158 ± 0.09	79.85
**G6 (1f)**	135.00	0.528 ± 0.02 *	32.66
**G7 (1g)**	125.00	0.463 ± 0.04	40.95
**G8 (1h)**	155.00	0.563 ± 0.14 *	28.19
**G9 (1i)**	175.00	0.698 ± 0.02	10.97
**G10 (1j)**	149.00	0.200 ± 0.06	74.49
**G11 (Ferulic acid)**	287.50	0.173 ± 0.08	77.94
**G12 (Diclofenac sodium)**	5.00	0.153 ± 0.10	89.49
**G 13 (Indomethacin)**	1.50	0.147 ± 0.02	81.25
**G14 (Tween 80)**	0.5 mL/100 g	0.784 ± 0.13	-

Data are presented as mean ± SD (*n* = 6). * *p* < 0.05 compared with the vehicle (Tween 80) control group.

**Table 5:** Liver function parameter values in rats in batches of *n* = 6 animals, 1 (**1a**), 2 (**1b**), 3 (**1c**), 4 (**1d**), 5 (**1e**), 6 (**1f**), 7 (**1g**), 8 (**1h**), 9 (**1i**), 10 (**1j**), 11 (**AF–ferulic acid**), 12 (**D-Diclofenac sodium**), 13 (**I-Indomethacin**), 14 (C1 inflammation, **Tween 80**) and 15 (C2 healthy, **Tween 80**) expressed as mean ± standard error of the mean (SEM).

Group/ Compound	AST (IU/L)	ALT (IU/L)	LDH (IU/L)	Total Bilirubin (mg/dL)	Direct Bilirubin (mg/dL)
**G1 (1a)**	244 ± 0.28	58 ± 0.56	2734.5 ± 0.34	0.11 ± 0.014	0.045 ± 0.007
**G2 (1b)**	124.5 ± 1.90	50.5 ± 0.63	617 ± 3.52	0.135 ± 0.01	0.025 ± 0.02
**G3 (1c)**	272.5 ± 0.63	96.5 ± 1.06	3778 ± 0.25	0.115 ± 0.01	0.035 ± 0.01
**G4 (1d)**	177.5 ± 0.07	40 ± 0.28	1824.5 ± 0.16	0.095 ± 0.01	0.035 ± 0.01
**G5 (1e)**	545 ± 7.70	130.5 ± 5.86	1181 ± 7.72	0.13 ± 0.09	0.045 ± 0.01
**G6 (1f)**	185.5 ± 5.72	52.5 ± 1.20	1866.5 ± 9.76	0.105 ± 0.01	0.03 ± 0.01
**G7 (1g)**	211 ± 0.28	56 ± 0.84	2450.5 ± 0.71	0.085 ± 0.01	0.006 ± 0.03
**G8 (1h)**	207.5 ± 1.62	67.5 ± 0.07	2046.5 ± 0.71	0.095 ± 0.02	0.003 ± 0.01
**G9 (1i)**	256.5 ± 0.21	69.5 ± 0.07	2555 ± 4.52	0.075 ± 0.03	0.055 ± 0.02
**G10 (1j)**	212.5 ± 1.48	70.5 ± 0.35	1826.5 ± 2.92	0.105 ± 0.01	0.045 ± 0.01
**G11 (AF)**	198.5 ± 2.47	65.5 ± 1.48	2020 ± 5.68	0.11 ± 0.01	0.055 ± 0.02
**G12 (D)**	133.5 ± 2.61	49 ± 0.56	1116 ± 6.15	0.115 ± 0.02	0.035 ± 0.01
**G13 (I)**	124.5 ± 0.35	59.5 ± 1.90	908.5 ± 4.16	0.175 ± 0.05	0.045 ± 0.01
**G14 (C1)**	269.5 ± 1.62	51 ± 1.41	1364 ± 4.78	0.115 ± 0.04	0.042 ± 0.01
**G15 (C2)**	100 ± 1.08	46.15 ± 1.33	400 ± 0.83	0.085 ± 0.04	0.03 ± 0.02

**Table 6:** Lipid profile parameter values in batch rats 1 (**1a**), 2 (**1b**), 3 (**1c**), 4 (**1d**), 5 (**1e**), 6 (**1f**), 7 (**1g**), 8 (**1h**), 9 (**1i**), 10 (**1j**), 11 (**AF–ferulic acid**), 12 (**D-diclofenac sodium**), 13 (**I-indomethacin**), 14 (C1 inflammation, **Tween 80**) and 15 (C2 healthy, **Tween 80**) expressed as ± standard error of the mean (SEM).

Group/ Compound	Total Cholesterol (mg/dL)	LDL Cholesterol (mg/dL)	HDL Cholesterol (mg/dL)	Triglycerides (mg/dL)
**G1 (1a)**	96 ± 2.12	13.5 ± 0.63	25 ± 0.28	66 ± 0.14
**G2 (1b)**	69.5 ± 0.77	10.5 ± 0.21	15 ± 0.56	63.5 ± 0.35
**G3 (1c)**	78 ± 0.56	8.5 ± 0.07	23.5 ± 0.35	95.5 ± 4.70
**G4 (1d)**	50 ± 0.28	7 ± 0.14	12 ± 0.28	66 ± 1.41
**G5 (1e)**	68 ± 0.84	6.5 ± 0.07	14.5 ± 0.07	142.5 ± 1.48
**G6 (1f)**	60 ± 1.27	6.5 ± 0.07	17.5 ± 0.49	98.5 ± 2.75
**G7 (1g)**	51.5 ± 1.06	6.5 ± 0.07	9.5 ± 0.35	72 ± 0.14
**G8 (1h)**	66 ± 0.28	7.5 ± 0.07	15 ± 0.28	85.5 ± 0.91
**G9 (1i)**	52 ± 0.84	6 ± 0.14	11.5 ± 0.35	61.5 ± 0.49
**G10 (1j)**	62.5 ± 1.62	6.5 ± 0.05	18.5 ± 0.63	77 ± 0.98
**G11 (AF)**	50.5 ± 0.21	8.5 ± 0.07	10.5 ± 0.21	53.5 ± 0.63
**G12 (D)**	67 ± 0.84	7.5 ± 0.70	18.5 ± 0.63	71 ± 0.98
**G13 (I)**	62.5 ± 0.21	10 ± 0.28	12.5 ± 0.07	78.5 ± 3.82
**G14 (C1)**	59 ± 0.14	8 ± 0.14	10.5 ± 0.07	53 ± 0.42
**G15 (C2)**	50.13 ± 1.08	10 ± 1.72	18.63 ± 0.72	54.25 ± 1.14

**Table 7:** Renal function parameter values in rats in groups 1 (**1a**), 2 (**1b**), 3 (**1c**), 4 (**1d**), 5 (**1e**), 6 (**1f**), 7 (**1g**), 8 (**1h**), 9 (**1i**), 10 (**1j**), 11 (**AF–ferulic acid**), 12 (**D-Diclofenac sodium**), 13 (**I-Indomethacin**), 14 (C1 inflammation, **Tween 80**) and 15 (C2 healthy, **Tween 80**) expressed as mean ± standard error of the mean (SEM).

Group/Compound	Urea (mg/dL)	Creatinine (mg/dL)	Uric Acid (mg/dL)
**G1 (1a)**	64.5 ± 1.90	0.58 ± 0.02	3.78 ± 0.05
**G2 (1b)**	42.5 ± 0.07	0.515 ± 0.03	1.905 ± 0.01
**G3 (1c)**	46 ± 0.42	0.60 ± 0.05	2.65 ± 0.04
**G4 (1d)**	43 ± 0.420	0.495 ± 0.007	2.61 ± 0.010
**G5 (1e)**	53 ± 0.70	0.58 ± 0.01	2.49 ± 0.05
**G6 (1f)**	34.5 ± 0.77	0.505 ± 0.02	1.80 ± 0.08
**G7 (1g)**	38.5 ± 0.07	0.47 ± 0.01	2.24 ± 0.04
**G8 (1h)**	49.5 ± 0.07	0.58 ± 0.02	3.59 ± 0.03
**G9 (1i)**	39.5 ± 0.070	0.515 ± 0.007	2.445 ± 0.016
**G10 (1j)**	32.5 ± 1.48	0.54 ± 0.04	2.23 ± 0.05
**G11 (AF)**	30.5 ± 0.070	0.545 ± 0.060	2.61 ± 0.066
**G12 (D)**	36.5 ± 0.630	0.46 ± 0.020	1.95 ± 0.010
**G13 (I)**	45.5 ± 0.77	0.49 ± 0.01	1.52 ± 0.03
**G14 (C1)**	35.5 ± 0.07	0.525 ± 0.01	2.24 ± 0.02
**G15 (C2)**	37.5 ± 1.97	0.63 ± 0.12	0.70 ± 0.18

**Table 8:** Values of hematological parameters determined in rats from groups 1 (**1a**), 2 (**1b**), 3 (**1c**), 4 (**1d**), 5 (**1e**), 6 (**1f**), 7 (**1g**), 8 (**1h**), 9 (**1i**), 10 (**1j**), 11 (**AF–ferulic acid**), 12 (**D-Diclofenac sodium**), 13 (**I-Indomethacin**), 14 (C1 inflammation, **Tween 80**) and 15 (C2 healthy, **Tween 80**) expressed as mean ± standard error of the mean (SEM).

Group/ Sample	Leukocytes (×10^3^/μL)	Erythrocytes (×10^6^/μL)	Hemoglobin (g/dL)	Hematocrit (%)	MCV (fL)	MCH (pg)	MCHC (g/dL)	Platelets (×10^3^/μL)
**G1 (1a)**	5.26 ± 0.03	7.93 ± 0.01	16.3 ± 0.24	45.7 ± 0.63	58 ± 0.26	20.63 ± 0.09	35.66 ± 0.04	1460 ± 2.03
**G2 (1b)**	14.63 ± 0.05	7.29 ± 0.04	14.73 ± 0.05	41.46 ± 0.05	57 ± 0.20	20.5 ± 0.02	36.26 ± 0.04	1131 ± 1.08
**G3 (1c)**	13.66 ± 0.25	7.05 ± 0.03	15.06 ± 0.03	41.73 ± 0.19	59.33 ± 0.20	21.36 ± 0.06	36.1 ± 0.12	1358 ± 1.71
**G4 (1d)**	12.2 ± 0.05	7.13 ± 0.06	15.23 ± 0.07	41.23 ± 0.22	58 ± 0.20	21.4 ± 0.08	36.96 ± 0.10	1280.33 ± 1.20
**G5 (1e)**	8.33 ± 0.04	5.35 ± 0.17	15.76 ± 0.40	33 ± 0.97	62.33 ± 0.47	17.4 ± 0.10	28.83 ± 0.06	1262 ± 5.67
**G6 (1f)**	13.33 ± 0.05	7.33 ± 0.05	14.9 ± 0.10	43.33 ± 0.26	61.00 ± 0.20	20.46 ± 0.02	34.06 ± 0.05	1442 ± 4.40
**G7 (1g)**	10.7 ± 0.21	7.73 ± 0.03	16.06 ± 0.07	42.7 ± 0.24	55 ± 0.10	20.8 ± 0.05	37.73 ± 0.12	1489 ± 1.92
**G8 (1h)**	7.43 ± 0.005	7.56 ± 0.17	15.36 ± 0.11	46.83 ± 0.16	64 ± 0.26	20.4 ± 0.02	32.06 ± 0.04	1258.66 ± 0.13
**G9 (1i)**	5.83 ± 0.11	7.14 ± 0.07	14.9 ± 0.11	40 ± 0.40	56 ± 0.10	20.86 ± 0.08	37.3 ± 0.10	1511.33 ± 2.75
**G10 (1j)**	15.46 ± 0.07	7.38 ± 0.15	14.06 ± 0.03	42.1 ± 0.10	64 ± 0.10	21.36 ± 0.04	33.4 ± 0.02	1105 ± 1.02
**G11 (AF)**	10.8 ± 0.59	7.7 ± 0.04	15.8 ± 0.03	43.9 ± 0.18	57 ± 0.26	20.5 ± 0.11	36.03 ± 0.09	1372.66 ± 2.09
**G12 (D)**	14.36 ± 0.69	6.74 ± 0.09	14.2 ± 0.15	40.1 ± 0.42	60 ± 0.52	21.2 ± 0.19	35.46 ± 0.05	1670.33 ± 1.48
**G13 (I)**	13.53 ± 0.70	5.81 ± 0.04	12.26 ± 0.08	33.96 ± 0.27	58.66 ± 0.30	21.16 ± 0.09	36.2 ± 0.05	1556.66 ± 1.03
**G14 (C1)**	9.76 ± 0.26	8.34 ± 0.04	16.46 ± 0.01	47.26 ± 0.06	56.66 ± 0.20	19.8 ± 0.09	34.86 ± 0.04	1209 ± 0.59
**G15 (C2)**	9.62 ± 0.10	8.51 ± 0.47	15.41 ± 1.57	43.13 ± 0.64	59.22 ± 2.63	17.05 ± 1.94	30.81 ± 2.04	1178 ± 0.56

MCV = mean corpuscular volume; MCH = mean corpuscular hemoglobin; MCHC = mean corpuscular hemoglobin concentration.

**Table 9:** Results of the biochemical parameters determined in the liver homogenate from the studied groups.

Group/Sample	MDA (nmol/g prot)	CAT (U/mg prot)	SOD (U/mg prot)	ALT (U/g prot)	AST (U/g prot)
**G1 (1a)**	18.61 ± 3.25	294.12 ± 9.50	271.95 ± 5.30	123.13 ± 7.65	121.12 ± 5.73
**G2 (1b)**	24.37 ± 5.53	286.17 ± 10.18	267.20 ± 6.42	116.04 ± 4.13	119.77 ± 3.37
**G3 (1c)**	23.78 ± 3,08	277.86 ± 11.87	258.46 ± 7.79	115.92 ± 6.88	116.53 ± 3.82
**G4 (1d)**	17.15 ± 1.78	291.42 ± 12.51	270.32 ± 6.89	127.46 ± 7.04	128.69 ± 4.31
**G5 (1e)**	25.55 ± 3.41	272.42 ± 9.81	265.58 ± 5.04	118.92 ± 5.69	120.89 ± 4.08
**G6 (1f)**	18.83 ± 2.96	291.37 ± 8.07	272.56 ± 5.62	126.43 ± 7.48	124.99 ± 5.74
**G7 (1g)**	17.56 ± 2.70	295.83 ± 9.02	273.39 ± 8.92	124.55 ± 6.10	126.11 ± 4.09
**G8 (1h)**	23.80 ± 3.11	277.55 ± 9.20	260.88 ± 7.55	117.50 ± 5.87	120.91 ± 5.26
**G9 (1i)**	20.50 ± 1.39	278.10 ± 13.80	264.93 ± 7.17	120.60 ± 8.76	121.97 ± 6.45
**G10 (1j)**	20.29 ± 3.95	290.55 ± 10.33	273.87 ± 5.02	124.79 ± 7.46	122.90 ± 6.61
**G 11 (C)**	14.74 ± 1.34	310.37 ± 10.71	282.08 ± 5.74	127.91 ± 3.73	129.23 ± 3.84
**G 12 (AF)**	14.90 ± 2.05	308.01 ± 9.17	277.85 ± 4.26	126.75 ± 5.59	127.04 ± 4.33
**G13 (D)**	15.63 ± 2.17	305.74 ± 8.51	279.71 ± 3.93	122.30 ± 5.25	124.02 ± 5.01
**G14 (I)**	15.09 ± 2.42	302.13 ± 10.40	276.49 ± 7.31	123.84 ± 4.95	116.17 ± 4.41

C = control (tween 80), AF = Ferulic acid, D = diclofenac sodium, I = Indomethacin.

**Table 10:** Structure of the rat groups and administered doses (mg/kg body weight) in the study of the anti-inflammatory effect using the acute inflammation model.

Group	Compound	Administered Dose (mg/kg b.w.)	Group	Compound	Administered Dose (mg/kg b.w.)
**1**	**1a**	98.75	**8**	**1h**	155
**2**	**1b**	159	**9**	**1i**	175
**3**	**1c**	481.25	**10**	**1j**	149
**4**	**1d**	92.5	**11**	**Ferulic acid**	287.5
**5**	**1e**	500	**12**	**Diclofenac sodium**	5
**6**	**1f**	135	**13**	**Indomethacin**	1.5
**7**	**1g**	125	**14**	**Tween 80**	0.5 mL/100 g

## Data Availability

The original contributions presented in this study are included in the article. Further inquiries can be directed to the corresponding author.
